# Parasitoid species diversity has no effect on protective symbiont diversity in experimental host‐parasitoid populations

**DOI:** 10.1002/ece3.11090

**Published:** 2024-03-07

**Authors:** Nina Hafer‐Hahmann, Christoph Vorburger

**Affiliations:** ^1^ EAWAG, Swiss Federal Institute of Aquatic Science and Technology Dübendorf Switzerland; ^2^ Department of Biology University of Konstanz Konstanz Germany; ^3^ Institute of Integrative Biology, ETH Zürich Zürich Switzerland

**Keywords:** adaptation, defensive symbiosis, experimental evolution, immune system, maintenance of diversity, specificity

## Abstract

How does diversity in nature come about? One factor contributing to this diversity are species interactions; diversity on one trophic level can shape diversity on lower or higher trophic levels. For example, parasite diversity enhances host immune diversity. Insect protective symbionts mediate host resistance and are, therefore, also engaged in reciprocal selection with their host's parasites. Here, we applied experimental evolution in a well‐known symbiont‐aphid‐parasitoid system to study whether parasitoid diversity contributes to maintaining symbiont genetic diversity. We used caged populations of black bean aphids (*Aphis fabae*), containing uninfected individuals and individuals infected with different strains of the bacterial endosymbiont *Hamiltonella defensa*, which protects aphids against parasitoids. Over multiple generations, these populations were exposed to three different species of parasitoid wasps (*Aphidius colemani*, *Binodoxys acalephae* or *Lysiphlebus fabarum*), simultaneous or sequential mixtures of these species or no wasps. Surprisingly, we observed little selection for *H. defensa* in most treatments, even when it clearly provided protection against a fatal parasitoid infection. This seemed to be caused by high induced costs of resistance: aphids surviving parasitoid attacks suffered an extreme reduction in fitness. In marked contrast to previous studies looking at the effect of different genotypes of a single parasitoid species, we found little evidence for a diversifying effect of multiple parasitoid species on symbiont diversity in hosts.

## Graphical abstract

Parasites play a crucial role in driving and maintaining the diversity of immune systems. Here we tested whether the diversity or parasites selects for the diversity of protective symbionts using an experimental evolution approach. Surprisingly, we found little selection for protective symbionts and little evidence for the diversifying effect of different parasitoid species.

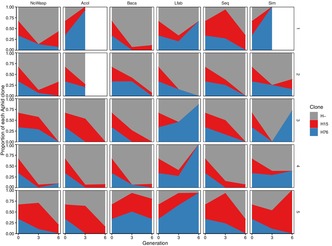

## 1 INTRODUCTION

How the diversity of living organisms in nature comes about is a question that has preoccupied ecologist and evolutionary biologists for centuries. Interactions between different organisms can play an important role in shaping this diversity (Chesson, 2000; Fine, 2015; Levine et al., 2017; Mcintire & Fajardo, 2014). More specifically, diversity on one trophic level—both within and between species—can shape and enhance diversity on higher and lower trophic levels (Cao et al., 2018; Dyer & Letourneau, 2003; Mailafiya et al., 2010; Morand, 2015). Similarly, the diversity of parasites has played an important role in the repeated diversification of immune systems of various organisms (Ghosh et al., 2011; Litman et al., 2007; Messier‐Solek et al., 2010). Protective symbionts—host associated (micro‐) organisms providing defence functions—can be considered from both perspectives. They can be seen as an additional line of defence complementing the host immune system and as such arguably being subject to similar selection pressures (Hafer & Vorburger, 2019) and as organisms in their own right from one trophic level that is affected by organisms from another trophic level, in this case the natural enemies against which they provide protection.

Protective symbionts are widespread throughout living organisms and are especially common in insects (Brownlie & Johnson, 2009; Flórez et al., 2015). Aphids represent one of the best‐studied systems, as they possess heritable bacterial endosymbionts providing effective protection against parasitoid wasps and pathogenic fungi (Guo et al., 2017; Oliver et al., 2014; Vorburger, 2014; Zytynska & Weisser, 2016). They do so in a very specific manner, whereby certain symbiont species provide protection against only a subset of the host's natural enemies (Asplen et al., 2014; Cayetano & Vorburger, 2015; Gimmi & Vorburger, 2024; Łukasik et al., 2013; McLean et al., 2020). There is also within‐species specificity, such that different strains of the same symbiont species provide unequal protection against different genotypes of the same parasitoid or pathogen (Cayetano et al., 2015; Cayetano & Vorburger, 2013, 2015; Leclair et al., 2016; McLean & Godfray, 2015; Parker et al., 2017; Rouchet & Vorburger, 2012; Schmid et al., 2012). The possession of resistance‐conferring symbionts often comes at fitness costs to the aphid host (Cayetano et al., 2015; Martinez et al., 2018; Sochard et al., 2019; Vorburger & Gouskov, 2011), albeit information is still lacking across parasitoid species, host genotypes and symbiont strains, limiting any general conclusions about these effects (Zytynska et al., 2021). Arguably, costs help to prevent any one symbiont from going to fixation in nature (Russell et al., 2013; Vorburger & Rouchet, 2016; Zytynska & Weisser, 2016). Symbionts are usually transmitted vertically with high fidelity (Darby & Douglas, 2003; Peccoud et al., 2014; Rock et al., 2018; Vorburger et al., 2017).

In the lab, strong reciprocal selection between parasitoids and symbionts has been shown repeatedly. Depending on the exact set up, it can result in the rapid fixation of the symbiont (Hafer‐Hahmann & Vorburger, 2020; Oliver et al., 2008; Rossbacher & Vorburger, 2020; Vorburger, 2014), extinction of the parasitoid (Käch et al., 2018) or an evolved ability of the parasitoid to overcome symbiont‐conferred resistance (Dennis et al., 2017; Dion et al., 2011; Rouchet & Vorburger, 2014). Two recent studies employing experimental evolution showed that parasitoid diversity can be crucial in maintaining symbiont diversity (Hafer‐Hahmann & Vorburger, 2020; Rossbacher & Vorburger, 2020). Both these studies focused on intraspecific variation, working with different strains of the well‐known aphid symbiont *Hamiltonella defensa* and different lines of the specialized parasitoid *Lysiphlebus fabarum*. Diversity in nature is certainly more extensive and involves not only intraspecific variation but also multiple parasitoid species attacking the same hosts (Müller et al., 1999; Van Veen et al., 2008). Two recent studies in natural populations observed that symbiont and parasitoid diversity were positively associated at the species level (Hafer‐Hahmann & Vorburger, 2021; Leclair et al., 2021). One possible explanation for this observation could be that species level diversity of parasitoids also plays a role in maintaining symbiont diversity. Here we tested experimentally whether species level parasitoid diversity has the potential to promote strain level diversity in a protective symbiont, using an experimental evolution approach.

## 2 METHODS

### 2.1 Insects

As a host we used the black bean aphid, *Aphis fabae*, an important pest of broad bean (*Vicia fabae*) and sugar beet (*Beta vulgaris*). We used three different clonal lines of the same *A. fabae* genotype which originated from a single female collected in St. Margrethen, Switzerland from *Chenopodium album* in 2006 (line A06‐405) and have been maintained clonally in the lab at 18–20°C and a 16/8 h light dark regime since. These lines differed only by their infections with the endosymbiotic bacterium *H. defensa*. *Hamiltonella defensa* serves as a protective symbiont in several aphid species, conferring resistance against parasitoid wasps (Asplen et al., 2014; Oliver et al., 2003, 2005; Rothacher et al., 2016; Schmid et al., 2012; Vorburger, 2014). Two of the aphid lines we used carried one of two genetically distinct *H. defensa* strains that had been introduced by microinjection (strain IDs H15 and H76) (Cayetano et al., 2015), while the third line was *H. defensa* free. We refer to these lines as H‐, H15 and H76 hereafter.

As parasitoids we used three different wasp species of the aphid‐specific subfamily Aphidiinae (Hymenoptera: Braconidae): *Lysiphlebus fabarum*, *Binodoxys acalephae* and *Aphidius colemani*. All are known to parasitize *A. fabae* in the field (Kavallieratos et al., 2013; Starý, 2006). *Lysiphlebus fabarum* occurs in sexual and asexual populations, while *B. acalephae* and *A. colemani* only reproduce sexually. To minimize differences between species, we used sexual *L. fabarum*. The sexual laboratory population was founded in 2012 by mixing nine independent accessions from six sites in Switzerland (Käch et al., 2018) and maintained since at high effective population size (500 individuals transferred every generation). The laboratory stock of *Binodoxys acalephae* was collected in 2018 near Zürich, Switzerland from *Aphis urticata*, and *A. colemani* was ordered from a commercial supplier (Andermatt Biocontrol, Grossdietwil, Switzerland). Since their collection/purchase and throughout the experiments, the wasps were reared on symbiont‐free *A. fabae* of a different clone than the one used in this study at ca. 22°C with a 16 h photoperiod.

### 2.2 Setup of the selection experiment

We followed a similar setup and protocol as we used previously for a related experiment manipulating intraspecific diversity of parasitoids (Hafer‐Hahmann & Vorburger, 2020). Briefly, we prepared 30 aphid populations comprising equal proportions of our three aphid lines (H‐, H15 and H76) in insect rearing cages (24.5 × 24.5 × 24.5 cm; BugDorm‐4F2222; MegaView Science, Taiwan). We placed three pots with 2‐weeks old broad bean plants (*Vicia faba*) that were inoculated with 9 adult females (3 per line) in every cage (27 aphids per cage). Within their cage aphids were able to move freely between plants. Treatments consisted either of no wasps (*NoWasp*), wasps of a single species (*A. colemani*, *B. acalephae* and *L. fabarum*, hereafter *Acol*, *Baca* and *Lfab*), a simultaneous mix with equal proportions of the three wasp species (*Sim*) or a sequential mix of the three wasp species (*Seq*), in which we applied a single species in each generation, but alternated species between generations (Generation 1 & 4: *B. acalephae*, generation 2 & 5: *A. colemani*, generation 3 & 6: *L. fabarum*). We set up five replicate cages per treatment. In the first generation, the wasp treatments were applied 5 days after the addition of the aphids, that is when the aphids had already produced small colonies of offspring. According to treatment we added either three females of *A. colemani* (the most virulent parasitoid), six females of *L. fabarum*, or six females of *B. acalaphae*, or a third of these numbers to each cage of treatment *Sim*. Due to low infection rates for *L. fabarum* and especially *B. acalephae*, we increased their number in the subsequent generations to 15 (5 for *Sim*) for *L. fabarum* and to 12–30 (4–10 for *Sim*) for *B. acalephae*. The numbers for this species were variable since we were not always able to obtain the desired number of wasps (see Table A1 for exact numbers). After adding the wasps to the cages, we left the populations undisturbed for 11 days to allow the wasps to attack and parasitize (i.e. kill and mummify) aphids, which take about 7–9 days after parasitoid oviposition to be recognizable as mummies. Parasitoids remained in the cage until they died naturally. Without additional food or water they only live for a few days (Ameri et al., 2015; Jerbi‐Elayed et al., 2021). After mummies had formed, we collected 30 healthy adult aphids from each cage, taking care to pick them from all plants and different parts of each plant and used them to establish the next aphid generation by inoculating new plants (10 aphids per plant) in fresh cages. If we were unable to obtain enough adult aphids, we substituted with the oldest nymphs we could find. Experimental evolution continued for 6 generations, although one replicate of *Acol* died out after the first generation and one replicate of the *NoWasp* treatment was contaminated with wasps during the second generation. These cages were replaced and set up anew with aphids from a different replicate of the same treatment. Three additional cages (two of *Acol*, one of *Sim*) died out in the last generation.

### 2.3 Data collection

During each transfer, we obtained the number of aphids (roughly estimated by counting aphids in groups of ca. 10 individuals) and mummies (exact counts) and plant size (i.e. total stem length of all plants) for each cage. We calculated mummification rate by dividing the number of mummies by the number of aphids plus mummies. To estimate aphid population composition (i.e. the relative frequencies of H‐, H15 and H76 aphids), we additionally collected 15 unmummified aphids per cage (5 aphids from each plant) at the end of the 3rd and last (6th), generation. These were stored at −20°C until further analysis.

We extracted aphid DNA using high salt extractions (Sunnucks & Hales, 1996), but adapted to a 96 deep well plate format (Gouskov et al., 2016; Hafer‐Hahmann & Vorburger, 2020). This DNA was then used for diagnostic PCRs (Ferrari et al., 2012) to test for the presence of *H. defensa* with a symbiont‐specific primer pair amplifying part of the bacterial 16S rRNA gene. Additionally, we amplified DNA of *Buchnera aphidicola* which, as an obligate symbiont of aphids, should be present in all individuals and hence served as a control for successful DNA extraction. 23 samples that were negative for *B. aphidicola* were discarded. PCRs were multiplexed for both symbionts using forward primer 16SA1 (AGAGTTTGATCMTGGCTCAG; Fukatsu & Nikoh, 1998) and reverse primer Buch_R_CV2 (CCCCCACTTTRGTTTTTCAAC; Hafer‐Hahmann & Vorburger, 2020) for *B. aphidicola* and forward primer 10F (AGTTTGATCATGGCTCAGATTG) and reverse primer T419R (AAATGGTATTCGCATTTATCG) for *H. defensa* (Ferrari et al., 2012). For each aphid possessing *H. defensa*, we additionally amplified part of *H. defensa*'s *murE* gene (forward primer: murE16F: ACTAACGGGAAAACCACTAATAC & reverse primer: murE936R: TTGAGAATGTCAGCGGTAATC); (Henry et al., 2013). This gene shows several sequence differences between *H. defensa* strains H15 and H76. Amplicons were sent to a commercial service (Microsynth, Balgach, Switzerland) for Sanger sequencing.

### 2.4 Infection experiments

In order to quantify the susceptibility of each aphid line to each parasitoid species, we used a fully crossed design to test each aphid line with each parasitoid species (between 14 and 27 replicates per combination). We conducted two rounds of this experiment, one before and one after the main experiment. For the first round, we set up replicates with two adult aphids each on one‐week‐old bean plants. Three to four days later, we removed the adults, counted the aphid nymphs and added wasps (2 females per plant), which remained on the plants until they died. Another 11 days later, we counted all non‐mummified aphids (i.e. surviving aphids and the offspring they had produced) and mummies. For the second round, we again added two aphids per plant, but removed them after 24 h. Two days thereafter, we counted the aphid nymphs and added two female wasps, which were removed after another 24 h. Ten to eleven days after the exposure to wasps, we counted aphids and mummies. We calculated mummification rate by dividing the number of mummies by the number of aphids when wasps were added. In order to estimate costs imposed by wasps not through direct mummification but through otherwise reducing aphid fitness, we calculated the number of offspring per non‐mummified aphid by dividing the number of aphids at the end of the experiment by the number of aphids when wasps were added minus the number of mummies. For the latter we excluded replicates in which no aphids survived the parasitoid attack. While we cannot rule out that some successfully parasitized aphids reached maturity prior to mummification, it seems unlikely that they contributed a meaningful number of offspring compared to healthy aphids.

### 2.5 Statistical analysis

We analysed data in R, version 4.0.0 (R Core Team, 2019) using linear mixed models from the *lme4* package (Bates et al., 2015). Plots were generated in ggplot2 (Wickham, 2016). Several of our response variables, especially estimates of proportions, showed strong overdispersion when analysed with generalized linear mixed models (GLMMs), resulting in unrealistic (anticonservative) *p*‐values. We, therefore, decided to use linear mixed models (LMMs) with variable transformations rather than GLMMs to obtain more realistic *p*‐values and to be able to use the same models across all response variables. For the experimental evolution experiment, we calculated symbiont haplotype number and Shannon diversity with *vegan* (Oksanen et al., 2019) and transformed our response variables if necessary to comply with model assumptions prior to building and testing statistical models. More precisely, we transformed mummy number, mummification rate, aphid number, number of different haplotypes and Shannon diversity using *transformTukey* from the package *rcompanion* (Mangiafico, 2019) to identify the best transformation which resulted in transforming data using a lambda of 0.325 (mummy number), 0.125 (mummification rate), 0.35 (aphid number), 1.325 (haplotype number) and 1.075 (Shannon). We transformed the proportion of H15 and H76 by using angular transformations of asin (*p* ^0.5^) and asin (*p* ^0.4^), respectively, to improve the normality of residuals. Plant size and the proportion of H‐ required no prior transformation. In each case the response variable in the experimental evolution experiment was a single value for each cage in each generation. Using each of these response variables, we fitted a separate LMM (lmer command) with cage ID as random effect and treatment, generation and their interaction as fixed effects. For a balanced design this model is equivalent to a repeated measures Anova. We defined contrasts as wasp presence versus wasp absence (not for mummification rate and mummy number), one versus multiple wasp species, multiple wasp species simultaneously (*Sim*) versus sequentially (*Seq*) and within single wasp treatments (*Acol*, *Baca*, *Lfab*). Each model was followed with a type III analysis of variance using Satterthwaite's method to obtain *p*‐values (Kuznetsova et al., 2017). Non‐significant interactions were removed from each model. Significant treatment effects or interactions were followed up with post hoc tests between each pairwise combination of treatments or between each treatment pair for each generation or between each pair of generations within each treatment using package *emeans* (Lenth, 2019) with Tukey corrections for multiple testing. In order to obtain beta‐diversity with confidence intervals we used *divEst* from package *entropart* and resampled 1000 times (Marcon & Hérault, 2015).

To analyse the experimental infections, similarly as described above, we used LMs followed by a type III analysis of variance and post hoc tests for significant effects with *emeans* (Lenth, 2019). We included aphid line (i.e. symbiont) and wasp species and their interaction as well as experimental round as fixed effects. To analyse aphid and mummy number at the end of the experiment, we included the number of aphids when adding wasps as a covariate. In order to confirm to model assumptions we used *transformTukey* for aphid and mummy number and the number of offspring per healthy aphid with a lambda of 0.275, 0.475 and 0.325, respectively, and an angular transformation of asin (*p* ^0.1^) for mummification rate.

## 3 RESULTS

### 3.1 Parasitism and changes in population composition

**FIGURE 1:**
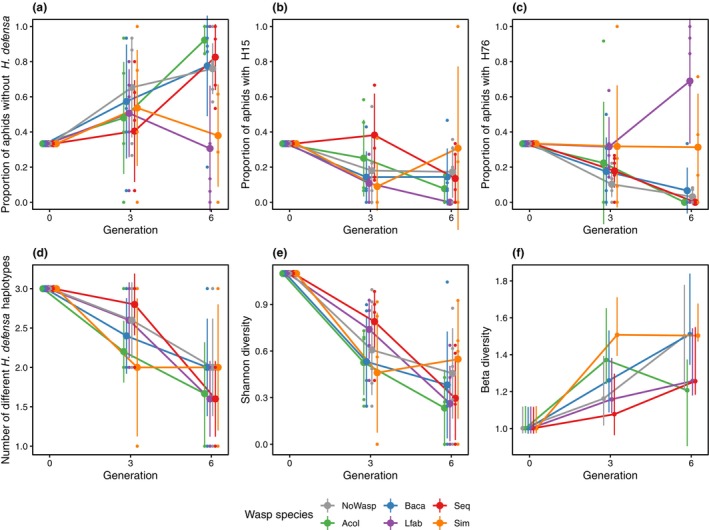
Proportion of aphids without *H. defensa* (a), with *H. defensa* haplotype H15 (b) and H76 (c) and *H. defensa* diversity (d: *H. defensa* strain richness; e: Shannon diversity; f: Beta‐diversity). Error bars represent 95% confidence intervals. Mean and confidence intervals for beta‐diversity have been calculated through bootstrapping.

**FIGURE 2:**
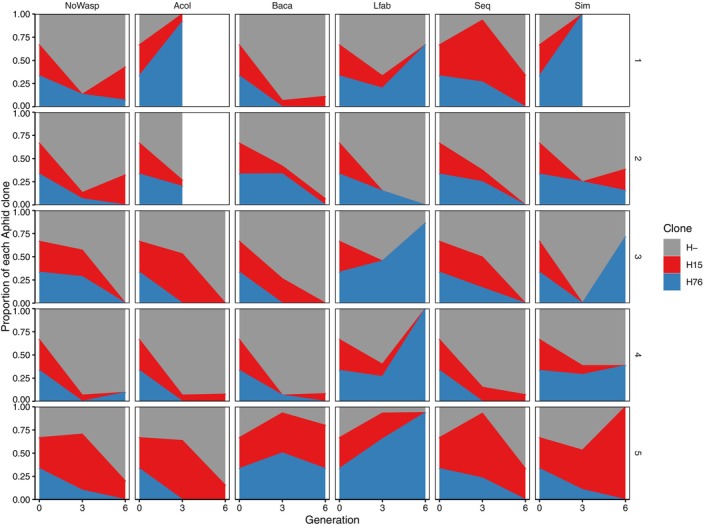
Proportion of aphids without *H. defensa* and with each *H. defensa* haplotype for each treatment and replicate. See Figure A1 for the same figure with the absolute number of aphids instead of proportions.

**FIGURE 3:**
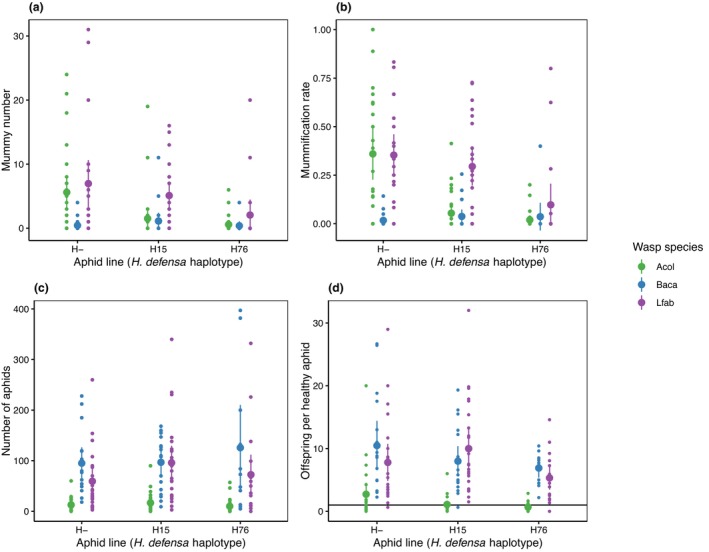
Outcome of experimental exposure tests. (a) Mummy number, (b) Mummification rate, (c) Aphid number at the end of the experiment, (d) Number of offspring per healthy aphid. Error bars represent 95% confidence intervals. Black line in panel d represents the number of offspring for a stable aphid population (i.e. *n* = 1).

Contrary to our expectation that infection with *H. defensa* would be beneficial in the presence of all parasitoids, it was the *H. defensa*‐free aphids that increased over time in all treatments except those that contained *L. fabarum* in every generation (*Lfab* and *Sim*, Figures 1a and 2, Table 1, Tables A2 and A3). That *H. defensa*‐infected aphids persisted in the presence of *L. fabarum* was mainly due to selection for aphids carrying H76, whereas H15‐infected aphids declined just as strongly as in the other treatments (Figures 1b,c and 2, Table 1, Tables A2 and A3). This was consistent with the results of our parasitism tests, in which H76 decreased mummification by *L. fabarum* significantly, whereas H15 did not (Figure 3a,b, Table 2, Tables A4 and A5). In the case of treatment *Baca*, the lack of selection for *H. defensa* was explicable by the generally low parasitism success of *B. acalephae* (Figure 3a,b). In parasitism tests, it produced an extremely low number of mummies even on *H. defensa*‐free aphids, such that there was no difference in parasitism among the three aphid lines used (Table 2, Tables A4 and A5). The same does not apply to treatment *Acol*. Both strains of *H. defensa* strongly reduced parasitism by *A. colemani* in our tests (Figure 3a,b), yet they declined in cages of the *Acol* treatment (Figure 1b,c). A noticeable difference in parasitism tests between *A. colemani* and the other parasitoids was an extremely low number of live aphids on plants (*p* < .001; Figure 3c, Table 2, Tables A4 and A5), reflecting a near‐absence of offspring from aphids that resisted parasitism (Figure 3c,d) compared to the other two wasp species (*p* < .001; Figure 3d, Table 2, Tables A4 and A5). This indicates strong negative effects of parasitoid attack/oviposition on host fitness even when the host does ultimately not succumb to the parasitoid (induced costs of resistance). This effect could be related to the fact that aphids went extinct in the last generation in two cages of the *Acol* treatment and one cage of the *Sim* treatment, even though the frequency of *H. defensa*‐protected aphids (especially with strain H76) was high before aphids were eradicated by parasitoids (Figure 2). Indeed, if anything, our experimental infection tests indicate high costs of H76, which reduced the number of offspring produced by non‐mummified aphids significantly (Figure 3d, Table 2, Tables A4 and A5) in the presence of any wasp species (*H. defensa* × wasp species interaction: *p* = .2337; Figure 3d, Table 2 and Table A4). When exposed to *A. colemani* this seems to have been especially detrimental; surviving adult aphids produced less than one offspring when carrying H76 (0.66 ± 0.27), resulting in negative growth, while it was just around 1 (1.08 ± 0.45) for aphids carrying H15 and usually well above 1 (2.72 ± 1.83) for aphids without *H. defensa*.

**TABLE 1:** Fixed effects tests from linear mixed effect models for the proportion of each aphid line in experimental populations and aphid line diversity.

Response	Factor	df	*F*	*p*
Proportion of aphids without *H. defensa*	Treatment	5, 23	1.25	.3175
	Generation	2, 46	13.95	**<.0001**
	Treatment × Generation	10, 46	2.11	**.0430**
Proportion of aphids with *H. defensa* haplotype H15	Treatment	5, 24	0.86	.5186
	Generation	2, 57	14.04	**<.0001**
	Treatment × Generation	10, 47	1.68	.1145
Proportion of aphids with *H. defensa* haplotype H76	Treatment	5, 20	4.25	**.0088**
	Generation	2, 42	11.55	**.0001**
	Treatment × Generation	10, 42	2.45	**.0208**
Number of different aphid lines (i.e. *H. defensa* strains)	Treatment	5, 79	0.49	.7832
	Generation	2, 79	40.81	**<.0001**
	Treatment × Generation	10, 69	0.96	.4825
Shannon diversity	Treatment	5, 79	0.30	.9119
	Generation	2, 79	65.74	**<.0001**
	Treatment × Generation	10, 69	0.97	.4811

*Note*: In order to obtain statistics for main effects if interactions were non‐significant, we build new models which did not contain the interactions. Statistics for interactions and main effects in models where the interaction was significant are from the full models. Significant *p*‐values have been highlighted in bold.

**TABLE 2:** Linear models for experimental exposures.

Response	Factor	df	*F*	*p*
Mummy number	Round	1, 171	42.43	**<.0001**
	Number of aphids prior to exposure	1, 171	58.42	**<.0001**
	*H. defensa*	2, 171	24.41	**<.0001**
	Wasp species	2, 171	26.16	**<.0001**
	*H. defensa* × Wasp species	4, 171	5.75	**.0002**
Mummification rate	Round	1, 172	15.49	**.0001**
	*H. defensa*	2, 172	25.77	**<.0001**
	Wasp species	2, 172	21.13	**<.0001**
	*H. defensa* × Wasp species	4, 172	4.96	**.0008**
Aphid number	Round	1, 175	13.00	**.0004**
	Number of aphids prior to exposure	1, 175	101.17	**<.0001**
	*H. defensa*	2, 175	3.78	**.0246**
	Wasp species	2, 175	106.11	**<.0001**
	*H. defensa* × Wasp species	4, 171	1.33	.2596
Offspring per non‐mummified aphid	Round	1, 174	8.24	**.0046**
	*H. defensa*	2, 174	7.57	**.0007**
	Wasp species	2, 174	86.02	**<.0001**
	*H. defensa* × Wasp species	4, 170	1.41	.2337

*Note*: In order to obtain statistics for main effects if interactions were non‐significant, we build new models which did not contain the interactions. Statistics for interactions and main effects in models where the interaction was significant are from the full models. Significant *p*‐values have been highlighted in bold.

### 3.2 Symbiont diversity

Arguably due to the prevailing selection against *H. defensa*, we saw no significant effect of parasitoid diversity on symbiont strain number or Shannon index (Figure 1d,e, Table 1 and Table A2). Also beta‐diversity, reflecting the variation in symbiont composition of cages from the same treatment, indicated no consistent differences between treatments with single (*Acol*, *Baca*, *Lfab*) and multiple (*Sim* and *Seq*) wasp species at the end of the cage experiment (Figure 1f, Table A6). The only and admittedly weak evidence for an effect of parasitoid diversity on symbiont diversity came from comparing the two treatments that maintained reasonably high levels of *H. defensa*, *Lfab* and *Sim*. For all measures of symbiont diversity (Figure 1d–f), treatment *Sim* with all three parasitoid species showed higher values than the treatment with *L. fabarum* only, which mainly selected for aphids infected with H76. There was only weak overlap of confidence intervals for beta‐diversity between these treatments (Figure 1f purple vs. orange line, Table A6).

### 3.3 Consequences for population dynamics

**FIGURE 4:**
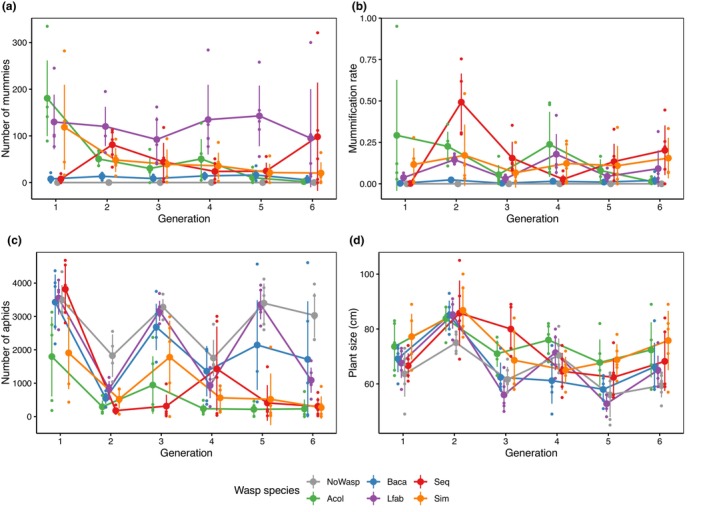
Population dynamics. (a) Mummy number, (b) Mummification rate, (c) Aphid number, (d) Plant size. Error bars represent 95% confidence intervals. Mean and confidence intervals for beta‐diversity have been calculated through bootstrapping.

We found no clear pattern of evolved resistance even in those treatments (*Lfab* and *Sim*) that showed some selection for *H. defensa*. Both, mummy number and mummification rate were significantly affected by treatment and this effect varied between generations, but the pattern showed no clear trends and this was paralleled by the number of aphids and plant size (Figure 4, Table 3, Tables A7 and A8). *Binodoxys acalephae* always showed very low numbers and rates of mummification and hence did not significantly diminish aphid numbers compared to cages without any wasps (*p* > .06; Figure 4, Table A8). By contrast, *A. colemani* produced a large number of mummies relative to aphid number and strongly reduced aphid numbers (*p* < .02; Figure 4, Table A8), with one cage going extinct within two generations and two out of five cages going extinct by the sixth generation. The third wasp species, *L. fabarum*, produced consistently high rates and numbers of mummies, but, similarly to *B. acalaphae*, this did not have a major impact on aphid numbers (*p* > .1; Figure 4, Table A8). The two treatments that received a mixture of wasps showed somewhat different patterns. In cages receiving different wasp species sequentially, the different species produced similar mummification as cages receiving the same wasp throughout (Figure 4, Table A8). Nevertheless, the numbers of aphids these cages harboured were high only in the first generation in which they had received the least aggressive wasp—*B. acalaphae*—but dropped in the subsequent generation and never clearly recovered thereafter (Figure 4, Table A8). Cages exposed to all three wasps simultaneously showed a similar, if less pronounced, pattern as those exposed only to the most aggressive wasp *A. colemani*: decreasing numbers of aphids but consistently high mummification rates (Figure 4, Table A8). One of these cages even went extinct.

**TABLE 3:** Fixed effects tests from linear mixed effect models for insect population dynamics and plant size.

Response	Factor	df	*F*	*p*
Mummy number	Treatment	4, 20	11.85	**<.0001**
	Generation	5, 100	5.82	**.0001**
	Treatment × Generation	20, 100	3.59	**<.0001**
Mummification rate	Treatment	4, 21	20.27	**<.0001**
	Generation	5, 94	11.62	**<.0001**
	Treatment × Generation	20, 94	4.34	**<.0001**
Aphid number	Treatment	5, 24	19.40	**<.0001**
	Generation	5, 119	23.91	**<.0001**
	Treatment × Generation	25, 119	2.66	**.0002**
Plant size	Treatment	5, 24	5.24	**.0021**
	Generation	5, 119	27.79	**<.0001**
	Treatment × Generation	25, 119	1.96	**.0085**

*Note*: Significant *p*‐values have been highlighted in bold.

## 4 DISCUSSION

Whether or not a heritable symbiont is maintained in a host population depends on its costs and benefits to the host. Both are often context‐dependent. Either strain of *H. defensa* we used here provided protection against fatal parasitoid infection: H76 against *A. colemani* and *L. fabarum*, H15 at least against *A. colemani* (see also Cayetano & Vorburger, 2015). Nevertheless, we saw strong selection against *H. defensa* in the presence of the parasitoids *A. colemani* and *B. acalephae*, as well as in the *Seq* treatment. The only parasitoid driving selection for aphids infected with *H. defensa*, specifically strain H76, was *L. fabarum*. This may also explain why *H. defensa* infections did not decline in the *Sim* treatment, where *L. fabarum* was continuously present in the parasitoid mixture. In this context it is important to note that *L. fabarum* is indeed the most common parasitoid of *A. fabae* in natural populations (Gimmi et al., 2023; Rothacher et al., 2016; Starý, 2006).

When it comes to costs of resistance, it is useful to distinguish the constitutive costs of possessing a defence mechanism and the induced costs of using this defence. This also applies to symbiont‐conferred defences (Vorburger et al., 2013). The well‐known constitutive costs of an infection with *H. defensa* (Cayetano et al., 2015; Martinez et al., 2018; Sochard et al., 2019; Vorburger & Gouskov, 2011) can explain why the prevalence of this symbiont declined in the absence of parasitoids and in the presence of the parasitoid *B. acalaphae*, which was so ineffective in parasitizing even *H. defensa*‐free aphids that it probably did not exert much selection for resistance. They cannot explain why aphids possessing *H. defensa* virtually disappeared in populations exposed to *A. colemani*. This wasp is an effective parasitoid and both strains of *H. defensa* strongly reduced parasitism by *A. colemani*. A likely explanation is provided by the high induced fitness costs of surviving an attack by *A. colemani*. The reproduction of aphids was strongly impaired if they survived parasitoid attack (i.e. survivors produced very few offspring), and this fitness reduction was stronger in the presence of *H. defensa*. Especially aphids carrying H76 suffered such a strong fitness cost that the resulting population growth was negative. In a different aphid species, *Myzus persicae*, *A. colemani* causes similarly severe induced costs in the presence of another protective symbiont, *Regiella insecticola* (Vorburger et al., 2008). By contrast, Vorburger et al. (2013) found no evidence for induced costs in *Aphis fabae* infected with *H. defensa* strain H76 (same as used here) after aphids survived oviposition by *L. fabarum*. This is consistent with the results from our parasitism tests here, where *H. defensa*‐infected survivors of exposures to *L. fabarum* still produced a reasonable amount of offspring, while those exposed to *A. colemani* did not (Figure 3c,d). Hence, there appears to be a clear difference in the effects the parasitoids we used have on *H. defensa*‐protected aphids, with *A. colemani* inducing severe costs even when it fails to parasitize the hosts successfully. In our study these costs seem to have been so strong in the presence of *A. colemani* that they were not sufficiently set off by benefits and hence aphids seem to have performed better by not paying these costs even if it left them vulnerable to parasitoid attack. Given that we worked with artificial symbiont‐aphid combinations generated by microinjection, it is fair to ask if these effects are representative of natural host‐symbiont associations. We believe that they are: both strains of *H. defensa* occur naturally in *A. fabae*, and because this aphid species reproduces sexually before overwintering (cyclical parthenogenesis), they find themselves in new host genotypes every year also in natural populations. Furthermore, Kaech et al. (2022) have shown that the fitness effects of natural and artificial combinations of *A. fabae* and *H. defensa* are very similar.

Parasitoid wasps and especially *Aphidius* spp. are frequently used as biological control for aphids in greenhouses. However, this control can be compromised by the presence of protective symbionts (Käch et al., 2018; Postic et al., 2020). Hence, from an applied point of view, using wasps that effectively select against protective symbionts could help to avoid this problem.

We have proposed that parasite and pathogen diversity could be an important driver in promoting and maintaining protective symbiont diversity (Hafer & Vorburger, 2019). Here we find, at best, very limited evidence for this hypothesis, mainly because we saw overall selection against the protective symbiont *H. defensa*, even in the presence of parasitoids against which it provides protection. However, these results were obtained in a very simplified laboratory environment. We cannot exclude that in much more complex natural environments, the interplay between induced costs, constitutive costs and benefits of protective symbionts could contribute to the variation in symbiont prevalence and composition we observe in natural populations. More support for the maintenance of symbiont diversity by parasitoids came from two earlier experiments, in which the genotypic composition of one parasitoid, *L. fabarum*, was manipulated rather than parasitoid species composition. These experiments provided clear evidence that genetically more diverse parasitoid populations can maintain higher strain diversity in *H. defensa* (Hafer‐Hahmann & Vorburger, 2020; Rossbacher & Vorburger, 2020). There was also strong selection in favour of *H. defensa*‐protected aphids and hence a high prevalence of the symbiont in those experiments, presumably providing more opportunity for any diversifying effects of parasitoid selection to come into play.

Our experiment only ran for six generations. While we cannot know how the prevalence of *H. defensa* (and its diversity) would have developed over a longer time period, the observed trends until generation six suggest that *H. defensa* would have gone extinct in all treatments in which *L. fabarum* was not present in each generation (treatments *nowasp*, *Baca*, *Acol* and *Seq*). We are convinced that the decline in *H. defensa* prevalence observed in these treatments is due to selection for *H. defensa*‐free aphids and not to vertical transmission failures, because under the laboratory conditions used here, maternal transmission is virtually perfect. Our stock cultures of these lines have meanwhile retained their infections for well over 10 years (C. Vorburger, personal observation). In the treatment with *L. fabarum* only—in agreement with previous findings (Hafer‐Hahmann & Vorburger, 2020; Rossbacher & Vorburger, 2020)—a single haplotype of H. defensa (H76) would likely have become fixed. The treatment using all parasitoids simultaneously seemed to induce the same trend, but it is less clear whether H76 would have trended towards fixation in all replicates. Only continuing the experiment for further generations would have been able to answer this question. However, by generation 6, the aphids had died out or were close to extinction in several cages, making it impossible to obtain more data.

Even though *H. defensa* is the best known protective symbiont in aphids, it is not the only one. Different species of symbionts seem to play a role in protecting aphids against parasitoids (Guo et al., 2017). Little is known about what maintains the coexistence and diversity of these different protective symbiont species. It is feasible that species level parasitoid diversity plays a role in maintaining their diversity, possibly more so than in maintaining strain diversity of *H. defensa*, as our largely negative results suggest. In support of this idea, two recent studies observed a positive association between symbiont and parasitoid species level diversity in natural aphid populations (Hafer‐Hahmann & Vorburger, 2021; Leclair et al., 2021). Even individual aphids can harbour multiple symbionts. Recent field data from the cereal aphid *Sitobion avenae* found a very high prevalence of co‐infections and suggests that rather than just individual symbionts certain symbiont combination could provide the best protection against particular parasitoids (Zytynska et al., 2023). In *A. fabae*, however, multiple infections do not appear to play an important role. Firstly, the prevalence of protective symbionts is generally lower in *A. fabae*, with *H. defensa* infecting approx. 30–40% of individuals in Central Europe (Gimmi et al., 2023). Secondly, co‐infections with *R. insecticola*, the second most abundant facultative endosymbiont, are less common than expected by chance (Gimmi et al., 2023; Vorburger & Rouchet, 2016), such that *H. defensa* typically occurs as single infections in *A. fabae*. For this reason, the protective effect of specific symbiont combination has never been tested experimentally in *A. fabae*.

There is mounting evidence that bottom‐up and top‐down effects across trophic levels maintain diversity within trophic levels (Cao et al., 2018; Dyer & Letourneau, 2003; Mailafiya et al., 2010; Morand, 2015). The diversity of plant communities, for example, has a positive effect on diversity in the soil and above ground spanning multiple trophic levels, albeit the strength of this effect decreases upwards in the food web (Scherber et al., 2010). Additionally, the diversity of soil communities (including pathogens and mutualists) and above ground insects can positively influence plant diversity (Bennett, 2010). It is tempting to propose that the same applies to the hidden level of symbiont communities within herbivorous insects—certainly an area that warrants further investigation.

## AUTHOR CONTRIBUTIONS

**Nina Hafer‐Hahmann:** Conceptualization (equal); formal analysis (lead); investigation (lead); writing – original draft (lead); writing – review and editing (equal). **Christoph Vorburger:** Conceptualization (equal); writing – original draft (supporting); writing – review and editing (equal).

## CONFLICT OF INTEREST STATEMENT

We have no conflicts of interest to disclose.

## Figures and Tables

**FIGURE A1:**
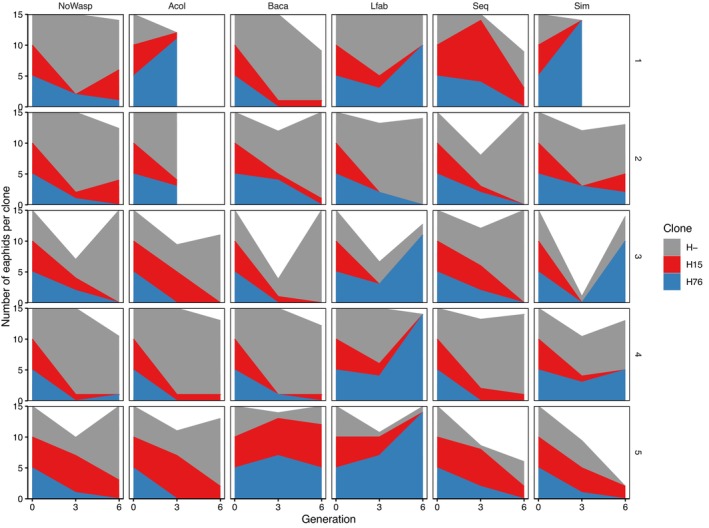
Number of aphids without *H. defensa* and with each *H. defensa* haplotype for each treatment and replicate. Note that at the beginning of the experiments (generation 0), we set up cages with 27 aphids, 9 per from each clonal line. For easier readability of the plot, we only included 15 aphids (5 per line) in the plot. Numbers of aphids not adding up to 15 in subsequent generations are either due to a failure to collect enough aphids or drop outs during molecular analysis.

## Data Availability

Data and code are available in the Dryad Digital Repository: https://doi.org/10.5061/dryad.s4mw6m9cm.

## APPENDIX 1

**TABLE A1:** Timing of experimental evolution experiment for each generation. Time is in days since setup/transfer. Transfer is for the previous/the subsequent generation, respectively.

Generation	Procedure	Time in days
1	Set up	0
1	Addition of wasps (*Acol*: 3/ *Baca*: 6/ *Lfab*: 6/ *Seq*: 6 *Baca*/ *Sim* 1/2/2)	5–6
1/2	Transfer	0/17–18
2	Addition of wasps (*Acol*: 3/ *Baca*: 30/ *Lfab*: 6/ *Seq*: 3 *Acol*/ *Sim* 1/10/5)	5
2/3	Transfer	0/16
3	Addition of wasps (*Acol*: 3/ *Baca*: 21/ *Lfab*: 6/ *Seq*: 15 *Lfab*/ *Sim* 1/7/5)	5
3/4	Transfer & sample	0/16
4	Addition of wasps (*Acol*: 3/ *Baca*: 24/ *Lfab*: 6/ *Seq*: 24 *Baca*/ *Sim* 1/8/5)	5
4/5	Transfer	0/16
5	Addition of wasps (*Acol*: 3/ *Baca*: 12–24/ *Lfab*: 6/ *Seq*: 3 *Acol*/ *Sim* 1/4–8/5)	5
5/6	Transfer	0/16
6	Addition of wasps (*Acol*: 3/ *Baca*: 12/ *Lfab*: 6/ *Seq*: 15 *Lfab*/ *Sim* 1/4/5)	5
6/7	Sample	0/16

**TABLE A2:** Summary of the best model in each case for aphid clone identity and diversity. Significant *p*‐values have been highlighted in bold.

Response	Random effect	Variance	SD
*Random effects*			
Proportion of aphids without *H. defensa*	Cage identity	0.01	0.11
	Residual	0.05	0.23
Proportion of aphids with *H. defensa* haplotype H15	Cage identity	0.01	0.10
	Residual	0.06	0.25
Proportion of aphids with *H. defensa* haplotype H76	Cage identity	0.01	0.09
	Residual	0.09	0.30
Number of different aphid lines (i.e. *H. defensa* strains)	Cage identity	<0.01	<0.01
	Residual	0.73	0.85
Shannon diversity	Cage identity	<0.01	<0.01
	Residual	0.07	0.26

Response	Fixed effect	Est.	SE	df	*t*	*p*
*Fixed effects*						
Proportion of aphids without *H. defensa*	(Intercept)	0.33	0.05	64	7.16	**<.0001**
	Treatment wasp presence vs. absence	<0.01	0.06	64	<0.01	1
	Treatment wasp number	<0.01	0.03	64	<0.01	1
	Treatment *Seq* vs. *Sim*	<0.01	0.08	64	<0.01	1
	Treatment *Acol* vs. *Baca*	<0.01	0.28	64	<0.01	1
	Treatment *Baca* vs. *Lfab*	<0.01	0.16	64	<0.01	1
	Generation 1 vs. 3	0.19	0.06	45	3.24	**.0022**
	Generation 1 vs. 6	0.32	0.06	46	5.21	**<.0001**

	Treatment wasp presence vs. absence: Generation 1 vs. 3	−0.05	0.08	45	−0.72	.4769
	Treatment wasp number: Generation 1 vs. 3	−0.04	0.04	45	−1.03	.3107
	Treatment *Seq* vs. *Sim*: Generation 1 vs. 3	−0.07	0.10	45	−0.64	.525
	Treatment *Acol* vs. *Baca*: Generation 1 vs. 3	0.23	0.36	45	0.64	.5275
	Treatment *Baca* vs. *Lfab*: Generation 1 vs. 3	0.15	0.21	45	0.71	.4792
	Treatment wasp presence vs. absence: Generation 1 vs. 6	−0.04	0.08	45	−0.47	.6384
	Treatment wasp number: Generation 1 vs. 6	−0.03	0.04	45	−0.93	.3553
	Treatment *Seq* vs. *Sim*: Generation 1 vs. 6	0.23	0.11	46	2.20	**.0330**
	Treatment *Acol* vs. *Baca*: Generation 1 vs. 6	0.44	0.36	45	1.23	.2247
Treatment *Baca* vs. *Lfab*: Generation 1 vs. 6	0.45	0.21	45	2.21	**.0323**
Proportion of aphids with *H. defensa* haplotype H15	(Intercept)	0.62	0.05	72	12.53	**<.0001**
	Treatment wasp presence vs. absence	−0.03	0.04	24	−0.67	.5063
	Treatment wasp number	0.01	0.02	24	0.29	.7744
	Treatment *Seq* vs. *Sim*	0.05	0.06	24	0.85	.404
	Treatment *Acol* vs. *Baca*	0.17	0.19	23	0.86	.3995
	Treatment *Baca* vs. *Lfab*	0.15	0.11	23	1.33	.1955
	Generation 1 vs. 3	−0.24	0.06	56	−3.68	**.0005**
	Generation 1 vs. 6	−0.34	0.07	57	−5.12	**<.0001**
Proportion of aphids with *H. defensa* haplotype H76	(Intercept)	0.70	0.06	68	12.45	**<.0001**
	Treatment wasp presence vs. absence	<0.01	0.07	68	<0.01	1
	Treatment wasp number	<0.01	0.03	68	<0.01	1
	Treatment *Seq* vs. *Sim*	<0.01	0.10	68	<0.01	1
	Treatment *Acol* vs. *Baca*	<0.01	0.34	68	<0.01	1
	Treatment *Baca* vs. *Lfab*	<0.01	0.19	68	<0.01	1
	Generation 1 vs. 3	−0.22	0.08	41	−2.89	**.0061**
	Generation 1 vs. 6	−0.38	0.08	43	−4.76	**<.0001**
	Treatment wasp presence vs. absence: Generation 1 vs. 3	0.05	0.10	41	0.46	.6464
	Treatment wasp number: Generation 1 vs. 3	0.04	0.05	41	0.85	.4024
	Treatment *Seq* vs. *Sim*: Generation 1 vs. 3	−0.09	0.13	41	−0.66	.5131
	Treatment *Acol* vs. *Baca*: Generation 1 vs. 3	−0.33	0.46	41	−0.73	.4719
	Treatment *Baca* vs. *Lfab*: Generation 1 vs. 3	−0.31	0.26	41	−1.18	.2434
	Treatment wasp presence vs. absence: Generation 1 vs. 6	0.12	0.10	42	1.16	.2532
	Treatment wasp number: Generation 1 vs. 6	0.03	0.05	41	0.65	.5216
	Treatment *Seq* vs. *Sim*: Generation 1 vs. 6	−0.30	0.14	43	−2.17	**.0356**
	Treatment *Acol* vs. *Baca*: Generation 1 vs. 6	−0.87	0.46	41	−1.90	.0640
	Treatment *Baca* vs. *Lfab*: Generation 1 vs. 6	−0.88	0.26	41	−3.32	**.0019**
Number of different aphid lines (i.e. *H. defensa* strains)	(Intercept)	4.29	0.16	79	27.5	**<.0001**
	Treatment wasp presence vs. absence	−0.11	0.12	79	−0.96	.3395
	Treatment wasp number	−0.04	0.05	79	−0.82	.4126
	Treatment *Seq* vs. *Sim*	0.12	0.16	79	0.78	.4392
	Treatment *Acol* vs. *Baca*	0.34	0.54	79	0.63	.5333
	Treatment *Baca* vs. *Lfab*	0.22	0.31	79	0.70	.4843
	Generation 1 vs. 3	−0.99	0.22	79	−4.50	**<.0001**
	Generation 1 vs. 6	−2.05	0.23	79	−9.03	**<.0001**
Shannon diversity	(Intercept)	1.11	0.05	79	23.64	**<.0001**
	Treatment wasp presence vs. absence	−0.03	0.03	79	−0.76	.4466
	Treatment wasp number	<0.01	0.02	79	−0.10	.9193
	Treatment *Seq* vs. *Sim*	0.02	0.05	79	0.38	.7034
	Treatment *Acol* vs. *Baca*	0.07	0.16	79	0.43	.6705
	Treatment *Baca* vs. *Lfab*	0.02	0.09	79	0.21	.8377
	Generation 1 vs. 3	−0.51	0.07	79	−7.75	**<.0001**
	Generation 1 vs. 6	−0.76	0.07	79	−11.15	**<.0001**

**TABLE A3:** Post hoc test to investigate significant interactions for aphid clone identity and diversity. Please note that generation 0 data consisted of the expected equal proportion set up at the beginning of the experiment. Hence, data for generation 0 has been omitted from the results for contrasts between treatment. Significant *p*‐values have been highlighted in bold.

Response	Treatment	Estimate 0	SE 0	Estimate 3	SE 3	Estimate 6	SE 6
*Estimates (estimated marginal means)*							
Proportion of aphids without *H. defensa*	*Acol*	0.33	0.114	0.48	0.114	0.91	0.147
	*Baca*	0.33	0.114	0.57	0.114	0.78	0.114
	*Lfab*	0.33	0.114	0.51	0.114	0.31	0.114
	*NoWasp*	0.33	0.114	0.65	0.114	0.76	0.114
	*Seq*	0.33	0.114	0.41	0.114	0.83	0.114
	*Sim*	0.33	0.114	0.54	0.114	0.36	0.127
Proportion of aphids with *H. defensa* haplotype H76	*Acol*	0.70	0.138	0.37	0.138	0.03	0.179
	*Baca*	0.70	0.138	0.38	0.138	0.14	0.138
	*Lfab*	0.70	0.138	0.67	0.138	1.03	0.138
	*NoWasp*	0.70	0.138	0.36	0.138	0.15	0.138
	*Seq*	0.70	0.138	0.46	0.138	0.00	0.138
	*Sim*	0.70	0.138	0.64	0.138	0.59	0.155

Response	Treatment	Generation	*z* 3	*p* 3	*z* 6	*p* 6
*Contrasts between generations for each treatment*						
Proportion of aphids without *H. defensa*	*Acol*	0	−1.01	.575	−3.35	**.004**
		3			−2.50	**.041**
	*Baca*	0	−1.65	.235	−3.05	**.011**
		3			−1.39	.353
	*Lfab*	0	−1.19	.464	0.19	.980
		3			1.38	.359
	*NoWasp*	0	−2.20	.082	−2.94	**.014**
		3			−0.73	.745
	*Seq*	0	−0.49	.875	−3.39	**.004**
		3			−2.89	**.016**
	*Sim*	0	−1.40	.350	−0.16	.986
		3			1.15	.491
Proportion of aphids with *H. defensa* haplotype H76	*Acol*	0	1.75	.197	3.07	**.009**
		3			1.57	.266
	*Baca*	0	1.73	.204	2.99	**.012**
		3			1.26	.425
	*Lfab*	0	0.16	.985	−1.74	.200
		3			−1.91	.148

	*NoWasp*	0	1.84	.169	2.96	**.013**
		3			1.12	.508
	*Seq*	0	1.27	.420	3.74	**.001**
		3			2.47	**.044**
	*Sim*	0	0.33	.940	0.53	.855
3				0.22	.974

Response	Contrast	*z* 3	*p* 3	*z* 6	*p* 6
*Contrasts between treatments for each generation*					
Proportion of aphids without *H. defensa*	*Acol*–*Baca*	−0.58	.992	0.73	.978
	*Acol*–*Lfab*	−0.17	1	3.26	**.021**
	*Acol*–*NoWasp*	−1.07	.890	0.81	.964
	*Acol*–*Seq*	0.46	.997	0.46	.997
	*Acol*–*Sim*	−0.35	.999	2.85	.062
	*Baca*–*Lfab*	0.41	.998	2.91	.053
	*Baca*–*NoWasp*	−0.50	.996	0.10	1
	*Baca*–*Seq*	1.04	.901	−0.31	1
	*Baca*–*Sim*	0.23	1	2.45	.156
	*Lfab*–*NoWasp*	−0.91	.943	−2.82	.068
	*Lfab*–*Seq*	0.63	.988	−3.22	**.023**
	*Lfab*–*Sim*	−0.19	1	−0.30	1
	*NoWasp*–*Seq*	1.54	.641	−0.41	.998
	*NoWasp*–*Sim*	0.72	.978	2.35	.188
	*Seq*–*Sim*	−0.82	.964	2.74	.081
Proportion of aphids with *H. defensa* haplotype H76	*Acol*–*Baca*	−0.02	1	−0.50	.996
	*Acol*–*Lfab*	−1.53	.649	−4.42	**<.001**
	*Acol*–*NoWasp*	0.08	1	−0.53	.995
	*Acol*–*Seq*	−0.47	.997	0.12	1
	*Acol*–*Sim*	−1.36	.749	−2.39	.173
	*Baca*–*Lfab*	−1.51	.660	−4.55	**<.001**
	*Baca*–*NoWasp*	0.10	1	−0.04	1
	*Baca*–*Seq*	−0.45	.998	0.72	.979
	*Baca*–*Sim*	−1.34	.760	−2.19	.256
	*Lfab*–*NoWasp*	1.61	.596	4.52	**<.001**
	*Lfab*–*Seq*	1.06	.895	5.27	**<.001**
	*Lfab*–*Sim*	0.16	1	2.09	.303
	*NoWasp*–*Seq*	−0.55	.994	0.75	.974
	*NoWasp*–*Sim*	−1.44	.700	−2.16	.272
	*Seq*–*Sim*	−0.90	.946	−2.87	.059

**TABLE A4:** Summary of best models for experimental exposures. Significant *p*‐values have been highlighted in bold.

Response	Fixed effect	Estimate	SE	*t*	*p*
Mummy number	(Intercept)	0.81	0.19	4.17	**<.001**
	Round after main experiment	−0.05	0.13	−0.36	.7157
	Number of aphids prior to exposure	0.04	0.01	7.31	**<.001**
	*H. defensa* H15	−1.05	0.19	−5.40	**<.001**
	*H. defensa* H76	−1.10	0.20	−5.50	**<.001**
	Wasp species *Baca*	−1.06	0.22	−4.79	**<.001**
	Wasp species *Lfab*	0.21	0.20	1.04	.2988
	*H. defensa* H15: Wasp species *Baca*	1.07	0.31	3.49	**.0006**
	*H. defensa* H76: Wasp species *Baca*	0.83	0.34	2.48	**.014**
	*H. defensa* H15: Wasp species *Lfab*	0.90	0.28	3.18	**.0017**
	*H. defensa* H76: Wasp species *Lfab*	−0.05	0.30	−0.16	.870
Mummification rate	(Intercept)	0.99	0.09	10.7	**<.001**
	Round after main experiment	−0.26	0.07	−3.96	**.0001**
	*H. defensa* H15	−0.56	0.12	−4.59	**<.001**
	*H. defensa* H76	−0.72	0.13	−5.71	**<.001**
	Wasp species *Baca*	−0.66	0.14	−4.73	**<.001**
	Wasp species *Lfab*	0.11	0.13	0.90	.3679
	*H. defensa* H15: Wasp species *Baca*	0.59	0.19	3.07	**.0025**
	*H. defensa* H76: Wasp species *Baca*	0.59	0.21	2.77	**.0063**
	*H. defensa* H15:Wasp species *Lfab*	0.46	0.18	2.56	**.0112**
	*H. defensa* H76: Wasp species *Lfab*	−0.05	0.19	−0.25	.8027
Aphid number	(Intercept)	0.71	0.17	4.31	**<.001**
	Round after main experiment	0.39	0.13	3.10	**.0023**
	Number of aphids prior to exposure	0.05	0.01	9.84	**<.001**
	*H. defensa* H15	0.15	0.11	1.35	.1774
	*H. defensa* H76	−0.09	0.12	−0.70	.4851
	Wasp species *Baca*	1.61	0.12	13.18	**<.001**
	Wasp species *Lfab*	1.23	0.11	11.11	**<.001**
Offspring per healthy aphid	(Intercept)	0.90	0.08	11.89	**<.001**
	Round after main experiment	0.15	0.07	2.25	**.0258**
	*H. defensa* H15	−0.02	0.08	−0.20	.8387
	*H. defensa* H76	−0.23	0.08	−2.84	**.0051**
	Wasp species *Baca*	0.92	0.08	11.15	**<.001**
	Wasp species *Lfab*	0.82	0.07	10.97	**<.001**

**TABLE A5:** Post hoc test to investigate significant interactions and main effects for experimental exposure tests. Significant *p*‐values have been highlighted in bold.

Response	Wasp species	Est. H‐	SE H‐	Est. H15	SE H15	Est. H76	SE H76
*Estimates (estimated marginal means)*							
Mummy number	*Acol*	1.42	0.14	0.38	0.14	0.32	0.14
	*Baca*	0.36	0.17	0.38	0.16	0.10	0.21
	*Lfab*	1.63	0.15	1.49	0.14	0.49	0.16
Mummification rate	*Acol*	0.86	0.09	0.30	0.09	0.14	0.09
	*Baca*	0.20	0.11	0.23	0.10	0.07	0.13
	*Lfab*	0.97	0.09	0.87	0.09	0.21	0.10
Aphid number	Overall	2.66	0.08	2.81	0.08	2.57	0.09
Offspring per non‐mummified aphid	Overall	1.55	0.06	1.54	0.05	1.32	0.06
		**Est. overall**			**SE overall**		
Aphid number	*Acol*	1.73			0.08		
	*Baca*	3.34			0.10		
	*Lfab*	2.96			0.08		
Offspring per non‐mummified aphid	*Acol*	0.89			0.05		
	*Baca*	1.81			0.06		
	*Lfab*	1.71			0.05		

Response	Contrast	*t* H‐	*p* H‐	*t* H15	*p* H15	*t* H76	*p* H76
*Contrasts between wasp species for each* H. defensa *treatment*							
Mummy number	*Acol*–*Baca*	4.79	**<.001**	−0.03	.999	0.89	.649
	*Acol*–*Lfab*	−1.04	.551	−5.61	**<.001**	−0.74	.738
	*Baca*–*Lfab*	−5.59	**<.001**	−5.08	**<.001**	−1.46	.312
Mummification rate	*Acol*–*Baca*	4.73	**<.001**	0.50	.872	0.45	.892
	*Acol*–*Lfab*	−0.90	.639	−4.59	**<.001**	−0.50	.873
	*Baca*–*Lfab*	−5.40	**<.001**	−4.66	**<.001**	−0.84	.676
		***t* Overall**			***p* Overall**		
Aphid number	*Acol*–*Baca*	−13.18			**<.001**		
	*Acol*–*Lfab*	−11.11			**<.001**		
	*Baca*–*Lfab*	3.01			**.008**		
Offspring per non‐mummified aphid	*Acol*–*Baca*	−11.15			**<.001**		
	*Acol*–*Lfab*	−10.97			**<.001**		
	*Baca*–*Lfab*	1.19			.460		

Response	Contrast	*t Acol*	*p Acol*	*t Baca*	*p Baca*	*t Lfab*	*p Lfab*
Contrasts between *H. defensa* treatments for each wasp species							
Mummy number	H‐ – H15	5.40	**<.001**	−0.08	.996	0.71	.757
	H‐ – H76	5.50	**<.001**	0.97	.596	5.21	**<.001**
	H15 – H76	0.26	.963	1.07	.534	4.59	**<.001**
Mummification rate	H‐ – H15	4.59	**<.001**	−0.23	.972	0.79	.707
	H‐ – H76	5.71	**<.001**	0.77	.724	5.53	**<.001**
	H15 – H76	1.28	.411	0.99	.585	4.83	**<.001**
		***t* Overall**			***p* Overall**		
Aphid number	H‐ – H15	−1.35			.367		
	H‐ – H76	0.07			.764		
	H15 – H76	1.97			.123		
Offspring per non‐mummified aphid	H‐ – H15	0.20			.977		
	H‐ – H76	2.84			**.014**		
	H15 – H76	2.70			**.021**		

**TABLE A6:** Calculated and estimated beta‐diversity. Estimated beta‐diversity and confidence intervals have been obtained through bootstrapping.

Treatment	Generation	Beta, measured	Beta, estimated	Lower CI	Upper CI
*NoWasp*	3	1.15	1.16	1.02	1.39
*Acol*	3	1.36	1.37	1.24	1.65
*Baca*	3	1.25	1.26	1.09	1.54
*Lfab*	3	1.15	1.16	1.08	1.38
*Seq*	3	1.07	1.08	0.97	1.31
*Sim*	3	1.50	1.51	1.39	1.71
*NoWasp*	6	1.50	1.50	1.21	1.60
*Acol*	6	1.20	1.21	0.90	1.37
*Baca*	6	1.50	1.51	1.35	1.85
*Lfab*	6	1.25	1.26	1.18	1.55
*Seq*	6	1.25	1.26	1.18	1.55
*Sim*	6	1.50	1.50	1.47	1.67

**TABLE A7:** Summary of the best model in each case for population dynamics. Significant *p*‐values have been highlighted in bold.

Response	Random effect	Variance	SD
*Random effects*			
Mummy Number	ID	0.17	0.42
	Residual	1.02	1.01
Mummification rate	ID	0000	0.02
	Residual	0.01	0.08
Aphid number	ID	1.34	1.16
	Residual	8.72	2.95
Plant size	ID	6.07	2.46
	Residual	59.71	7.73

Response	Fixed effect	Estimate	SE	df	*t*	*p*
*Fixed effects*						
Mummy Number	(Intercept)	3.56	0.22	108	16.27	**<.0001**
	Treatment wasp number	−0.19	0.09	108	−2.17	**.0325**
	Treatment *Seq* vs. *Sim*	−1.37	0.35	108	−3.96	**.0001**
	Treatment *Acol* vs. *Baca*	1.35	0.40	108	3.39	**.0010**
	Treatment *Baca* vs. *Lfab*	−0.82	0.40	108	−2.05	**.0424**
	Generation 1 vs. 2	0.04	0.29	100	0.14	.8910
	Generation 1 vs. 3	−0.65	0.29	100	−2.26	**.0260**
	Generation 1 vs. 4	−0.45	0.29	100	−1.56	.1210
	Generation 1 vs. 5	−0.82	0.29	100	−2.85	**.0053**
	Generation 1 vs. 6	−1.23	0.29	100	−4.29	**<.0001**
	Treatment wasp number: Generation 1 vs. 2	0.24	0.12	100	2.08	**.0399**
	Treatment *Seq* vs. *Sim*: Generation 1 vs. 2	1.72	0.45	100	3.79	**.0003**
	Treatment *Acol* vs. *Baca*: Generation 1 vs. 2	−1.30	0.52	100	−2.49	**.0144**
	Treatment *Baca* vs. *Lfab*: Generation 1 vs. 2	−0.36	0.52	100	−0.68	.4980
	Treatment wasp number: Generation 1 vs. 3	0.20	0.12	100	1.72	.0882
	Treatment *Seq* vs. *Sim*: Generation 1 vs. 3	1.41	0.45	100	3.12	**.0024**
	Treatment *Acol* vs. *Baca*: Generation 1 vs. 3	−1.71	0.52	100	−3.28	**.0014**
	Treatment *Baca* vs. *Lfab*: Generation 1 vs. 3	−0.49	0.52	100	−0.93	.3537

	Treatment wasp number: Generation 1 vs. 4	0.02	0.12	100	0.20	.8418
	Treatment *Seq* vs. *Sim*: Generation 1 vs. 4	1.06	0.45	100	2.34	**.0211**
	Treatment *Acol* vs. *Baca*: Generation 1 vs. 4	−1.58	0.52	100	−3.02	**.0032**
	Treatment *Baca* vs. *Lfab*: Generation 1 vs. 4	−0.51	0.52	100	−0.98	.3288
	Treatment wasp number: Generation 1 vs. 5	0.08	0.12	100	0.72	.4720
	Treatment *Seq* vs. *Sim*: Generation 1 vs. 5	1.55	0.45	100	3.43	**.0009**
	Treatment *Acol* vs. *Baca*: Generation 1 vs. 5	−2.66	0.52	100	−5.09	**<.0001**
	Treatment *Baca* vs. *Lfab*: Generation 1 vs. 5	−1.11	0.52	100	−2.12	**.0363**
	Treatment wasp number: Generation 1 vs. 6	0.31	0.12	100	2.64	**.0095**
	Treatment *Seq* vs. *Sim*: Generation 1 vs. 6	2.02	0.45	100	4.46	**<.0001**
	Treatment *Acol* vs. *Baca*: Generation 1 vs. 6	−2.58	0.52	100	−4.94	**<.0001**
Treatment *Baca* vs. *Lfab*: Generation 1 vs. 6	−0.89	0.52	100	−1.71	.0898
Mummification rate	(Intercept)	0.61	0.02	109	35.33	**<.0001**
	Treatment wasp number	−0.01	0.01	109	−1.37	.1747
	Treatment *Seq* vs. *Sim*	−0.12	0.03	109	−4.26	**<.0001**
	Treatment *Acol* vs. *Baca*	0.16	0.03	109	5.23	**<.0001**
	Treatment *Baca* vs. *Lfab*	−0.02	0.03	109	−0.75	.4535
	Generation 1 vs. 2	0.17	0.02	93	7.12	**<.0001**
	Generation 1 vs. 3	0.03	0.02	93	1.44	.1518
	Generation 1 vs. 4	0.09	0.02	92	3.69	**.0004**
	Generation 1 vs. 5	0.07	0.02	94	2.77	**.0067**
	Generation 1 vs. 6	0.07	0.02	96	2.98	**.0037**
	Treatment wasp number: Generation 1 vs. 2	0.03	0.01	93	2.89	**.0049**
	Treatment *Seq* vs. *Sim*: Generation 1 vs. 2	0.20	0.04	94	5.19	**<.0001**
	Treatment *Acol* vs. *Baca*: Generation 1 vs. 2	−0.08	0.04	92	−1.98	.0504
	Treatment *Baca* vs. *Lfab*: Generation 1 vs. 2	−0.02	0.04	92	−0.41	.6819
	Treatment wasp number: Generation 1 vs. 3	0.03	0.01	93	3.40	**.0010**
	Treatment *Seq* vs. *Sim*: Generation 1 vs. 3	0.18	0.04	94	4.83	**<.0001**
	Treatment *Acol* vs. *Baca*: Generation 1 vs. 3	−0.08	0.04	94	−1.71	.0914
	Treatment *Baca* vs. *Lfab*: Generation 1 vs. 3	−0.01	0.04	93	−0.19	.8467
	Treatment wasp number: Generation 1 vs. 4	0.00	0.01	92	−0.11	.9144
	Treatment *Seq* vs. *Sim*: Generation 1 vs. 4	0.08	0.04	92	2.14	**.0350**
	Treatment *Acol* vs. *Baca*: Generation 1 vs. 4	−0.09	0.04	92	−2.04	**.0444**
	Treatment *Baca* vs. *Lfab*: Generation 1 vs. 4	−0.04	0.04	92	−1.00	.3204
	Treatment wasp number: Generation 1 vs. 5	0.03	0.01	94	3.17	**.0020**
	Treatment *Seq* vs. *Sim*: Generation 1 vs. 5	0.12	0.04	95	3.13	**.0023**
	Treatment *Acol* vs. *Baca*: Generation 1 vs. 5	−0.10	0.04	94	−2.28	**.0250**
	Treatment *Baca* vs. *Lfab*: Generation 1 vs. 5	−0.01	0.04	93	−0.16	.8747
	Treatment wasp number: Generation 1 vs. 6	0.05	0.01	96	4.68	**<.0001**
	Treatment *Seq* vs. *Sim*: Generation 1 vs. 6	0.15	0.04	97	3.76	**.0003**
	Treatment *Acol* vs. *Baca*: Generation 1 vs. 6	−0.19	0.05	97	−4.03	**.0001**
	Treatment *Baca* vs. *Lfab*: Generation 1 vs. 6	−0.06	0.04	93	−1.32	.1916
Aphid number	(Intercept)	15.96	0.58	131	27.55	**<.0001**
	Treatment wasp presence vs. absence	−0.70	0.74	131	−0.95	.3456
	Treatment wasp number	−0.35	0.35	131	−1.01	.3165
	Treatment *Seq* vs. *Sim*	2.24	1.00	131	2.23	**.0274**
	Treatment *Acol* vs. *Baca*	0.14	3.48	131	0.04	.9682
	Treatment *Baca* vs. *Lfab*	−0.08	2.01	131	−0.04	.9685
	Generation 1 vs. 2	−6.88	0.77	120	−8.92	**<.0001**
	Generation 1 vs. 3	−3.00	0.76	119	−3.93	**.0001**
	Generation 1 vs. 4	−5.67	0.76	119	−7.44	**<.0001**
	Generation 1 vs. 5	−4.80	0.76	119	−6.30	**<.0001**
	Generation 1 vs. 6	−6.82	0.76	119	−8.94	**<.0001**
	Treatment wasp presence vs. absence: Generation1 vs. 2	−1.35	1.02	121	−1.32	.1887
	Treatment wasp number: Generation 1 vs. 2	−0.95	0.48	121	−1.99	**.0485**
	Treatment *Seq* vs. *Sim*: Generation 1 vs. 2	−3.47	1.32	119	−2.63	**.0096**
	Treatment *Acol* vs. *Baca*: Generation 1 vs. 2	7.99	4.78	121	1.67	.0969
	Treatment *Baca* vs. *Lfab*: Generation 1 vs. 2	3.44	2.73	121	1.26	.2098
	Treatment wasp presence vs. absence: Generation1 vs. 3	−0.22	0.98	119	−0.23	.8190
	Treatment wasp number: Generation 1 vs. 3	−1.20	0.46	119	−2.63	**.0096**
	Treatment *Seq* vs. *Sim*: Generation 1 vs. 3	−5.01	1.32	119	−3.80	**.0002**
	Treatment *Acol* vs. *Baca*: Generation 1 vs. 3	1.34	4.57	119	0.29	.7696
	Treatment *Baca* vs. *Lfab*: Generation 1 vs. 3	0.29	2.64	119	0.11	.9122
	Treatment wasp presence vs. absence: Generation1 vs. 4	−0.96	0.98	119	−0.99	.3257
	Treatment wasp number: Generation 1 vs. 4	−0.44	0.46	119	−0.95	.3421
	Treatment *Seq* vs. *Sim*: Generation 1 vs. 4	−1.27	1.32	119	−0.96	.3379
	Treatment *Acol* vs. *Baca*: Generation 1 vs. 4	4.38	4.57	119	0.96	.3406
	Treatment *Baca* vs. *Lfab*: Generation 1 vs. 4	2.94	2.64	119	1.11	.2679
	Treatment wasp presence vs. absence: Generation1 vs. 5	−1.16	0.98	119	−1.19	.2374
	Treatment wasp number: Generation 1 vs. 5	−1.72	0.46	119	−3.75	**.0003**
	Treatment *Seq* vs. *Sim*: Generation 1 vs. 5	−2.24	1.32	119	−1.70	.0918
	Treatment *Acol* vs. *Baca*: Generation 1 vs. 5	3.16	4.57	119	0.69	.4909
	Treatment *Baca* vs. *Lfab*: Generation 1 vs. 5	0.12	2.64	119	0.05	.9632
	Treatment wasp presence vs. absence: Generation1 vs. 6	−2.35	0.98	119	−2.40	**.0178**
	Treatment wasp number: Generation 1 vs. 6	−1.78	0.46	119	−3.89	**.0002**
	Treatment *Seq* vs. *Sim*: Generation 1 vs. 6	−1.46	1.32	119	−1.11	.2699
	Treatment *Acol* vs. *Baca*: Generation 1 vs. 6	10.6	4.57	119	2.32	**.0222**
	Treatment *Baca* vs. *Lfab*: Generation 1 vs. 6	5.14	2.64	119	1.95	.0539
Plant size	(Intercept)	69.63	1.48	137	47.03	**<.0001**
	Treatment wasp presence vs. absence	2.71	1.90	137	1.43	.1547
	Treatment wasp number	1.66	0.89	137	1.87	.0638
	Treatment *Seq* vs. *Sim*	−5.30	2.56	137	−2.07	**.0407**
	Treatment *Acol* vs. *Baca*	−9.60	8.88	137	−1.08	.2818
	Treatment *Baca* vs. *Lfab*	−4.00	5.13	137	−0.78	.4368
	Generation 1 vs. 2	13.98	2.02	120	6.93	**<.0001**
	Generation 1 vs. 3	−3.03	2.00	119	−1.52	.1311
	Generation 1 vs. 4	−1.53	2.00	119	−0.77	.4437
	Generation 1 vs. 5	−8.67	2.00	119	−4.34	**<.0001**
	Generation 1 vs. 6	−1.80	2.00	119	−0.90	.3688
	Treatment wasp presence vs. absence: Generation1 vs. 2	1.33	2.67	121	0.50	.6192
	Treatment wasp number: Generation 1 vs. 2	0.58	1.25	121	0.47	.6415
	Treatment *Seq* vs. *Sim*: Generation 1 vs. 2	4.80	3.46	119	1.39	.1674
	Treatment *Acol* vs. *Baca*: Generation 1 vs. 2	−10.43	12.49	121	−0.84	.4053
	Treatment *Baca* vs. *Lfab*: Generation 1 vs. 2	−6.12	7.14	121	−0.86	.3933
	Treatment wasp presence vs. absence: Generation1 vs. 3	−2.79	2.55	119	−1.09	.2765
	Treatment wasp number: Generation 1 vs. 3	0.88	1.20	119	0.74	.4637
	Treatment *Seq* vs. *Sim*: Generation 1 vs. 3	1.10	3.46	119	3.18	**.0019**
	Treatment *Acol* vs. *Baca*: Generation 1 vs. 3	14.4	11.97	119	1.20	.2314
	Treatment *Baca* vs. *Lfab*: Generation 1 vs. 3	9.60	6.91	119	1.39	.1674
	Treatment wasp presence vs. absence: Generation1 vs. 4	−2.77	2.55	119	−1.09	.2799
	Treatment wasp number: Generation 1 vs. 4	−2.78	1.20	119	−2.32	**.0219**
	Treatment *Seq* vs. *Sim*: Generation 1 vs. 4	5.10	3.46	119	1.48	.1426
	Treatment *Acol* vs. *Baca*: Generation 1 vs. 4	17.8	11.97	119	1.49	.1397
	Treatment *Baca* vs. *Lfab*: Generation 1 vs. 4	3.00	6.91	119	0.43	.6650
	Treatment wasp presence vs. absence: Generation1 vs. 5	−1.59	2.55	119	−0.62	.5358
	Treatment wasp number: Generation 1 vs. 5	0.26	1.20	119	0.22	.8284
	Treatment *Seq* vs. *Sim*: Generation 1 vs. 5	2.10	3.46	119	0.61	.5445
	Treatment *Acol* vs. *Baca*: Generation 1 vs. 5	10.8	11.97	119	0.90	.3688
	Treatment *Baca* vs. *Lfab*: Generation 1 vs. 5	7.20	6.91	119	1.04	.2996
	Treatment wasp presence vs. absence: Generation1 vs. 6	0.68	2.55	119	0.27	.7906
	Treatment wasp number: Generation 1 vs. 6	0.86	1.20	119	0.72	.4739
	Treatment *Seq* vs. *Sim*: Generation 1 vs. 6	1.50	3.46	119	0.43	.6650
	Treatment *Acol* vs. *Baca*: Generation 1 vs. 6	−2.80	11.97	119	−0.23	.8155
	Treatment *Baca* vs. *Lfab*: Generation 1 vs. 6	−1.60	6.91	119	−0.23	.8173

**TABLE A8:** Post hoc test to investigate significant interactions for population dynamics. Significant *p*‐values have been highlighted in bold.

Response	Treatment	Estimate1	SE1	Estimate2	SE2	Estimate3	SE3	Estimate4	SE4	Estimate5	SE5	Estimate6	SE6
*Estimates (estimated marginal means)*													
Mummy number	*Acol*	5.30	0.490	3.55	0.490	2.54	0.490	3.23	0.490	1.66	0.490	0.87	0.490
	*Baca*	1.77	0.490	2.27	0.490	1.95	0.490	2.35	0.490	2.34	0.490	1.62	0.490
	*Lfab*	4.77	0.490	4.68	0.490	4.21	0.490	4.79	0.490	4.89	0.490	3.82	0.490
	*Seq*	1.61	0.490	4.09	0.490	2.98	0.490	2.29	0.490	2.60	0.490	3.33	0.490
	*Sim*	4.35	0.490	3.40	0.490	2.90	0.490	2.91	0.490	2.24	0.490	2.03	0.490
Mummification rate	*Acol*	0.79	0.038	0.82	0.038	0.69	0.042	0.80	0.038	0.70	0.042	0.58	0.049
	*Baca*	0.45	0.038	0.63	0.038	0.48	0.038	0.58	0.038	0.55	0.038	0.56	0.038
	*Lfab*	0.66	0.038	0.78	0.038	0.63	0.038	0.79	0.038	0.67	0.038	0.69	0.038
	*Seq*	0.46	0.042	0.91	0.038	0.78	0.038	0.63	0.042	0.75	0.038	0.83	0.042
	*Sim*	0.70	0.038	0.76	0.038	0.66	0.038	0.71	0.038	0.74	0.042	0.77	0.042
Aphid number	*Acol*	12.59	1.419	6.95	1.419	9.81	1.419	6.47	1.419	5.15	1.419	4.64	1.419
	*Baca*	17.14	1.419	8.97	1.419	15.72	1.419	11.87	1.419	13.89	1.419	10.77	1.419
	*Lfab*	17.44	1.419	10.38	1.419	16.78	1.419	10.66	1.419	17.11	1.419	11.38	1.419
	*NoWasp*	17.36	1.419	13.74	1.580	16.99	1.419	13.52	1.419	17.15	1.419	16.45	1.419
	*Seq*	17.85	1.419	5.99	1.419	6.45	1.419	10.56	1.419	6.81	1.419	6.58	1.419
	*Sim*	13.38	1.419	8.46	1.419	12.01	1.419	8.63	1.419	6.83	1.419	5.03	1.419
Plant size	*Acol*	73.60	3.627	83.80	3.627	71.00	3.627	76.00	3.627	67.80	3.627	72.40	3.627
	*Baca*	69.20	3.627	85.00	3.627	62.40	3.627	61.20	3.627	58.00	3.627	66.20	3.627
	*Lfab*	67.60	3.627	85.20	3.627	56.00	3.627	71.40	3.627	52.80	3.627	65.00	3.627
	*NoWasp*	63.60	3.627	75.08	4.050	61.60	3.627	70.40	3.627	56.00	3.627	59.40	3.627
	*Seq*	66.60	3.627	85.80	3.627	80.00	3.627	64.60	3.627	62.40	3.627	68.20	3.627
	*Sim*	77.20	3.627	86.80	3.627	68.60	3.627	65.00	3.627	68.80	3.627	75.80	3.627

Response	Treatment	Generation	*z*2	*p*2	*z*3	*p*3	*z*4	*p*4	*z*5	*p*5	*z*6	*p*6
*Contrasts between generations for each treatment*												
Mummy number	*Acol*	1	2.73	.078	4.32	**.001**	3.24	**.020**	5.69	**<.001**	6.92	**<.001**
	*Acol*	2			1.58	.612	0.51	.996	2.96	**.043**	4.19	**.001**
	*Acol*	3					−1.08	.890	1.38	.741	2.60	.106
	*Acol*	4							2.45	.149	3.68	**.005**
	*Acol*	5									1.23	.822
	*Baca*	1	−0.78	.970	−0.28	1	−0.89	.947	−0.88	.950	0.24	1
	*Baca*	2			0.50	.996	−0.11	1	−0.10	1	1.02	.909
	*Baca*	3					−0.62	.989	−0.61	.990	0.52	.995
	*Baca*	4							0.01	1	1.14	.865
	*Baca*	5									1.13	.870
	*Lfab*	1	0.14	1	0.88	.951	−0.03	1	−0.19	1	1.48	.675
	*Lfab*	2			0.74	.977	−0.17	1	−0.34	.999	1.34	.762
	*Lfab*	3					−0.91	.944	−1.07	.891	0.61	.990
	*Lfab*	4							−0.17	1	1.51	.657
	*Lfab*	5									1.68	.549
	*Seq*	1	−3.88	**.002**	−2.14	.277	−1.07	.893	−1.55	.635	−2.68	.088
	*Seq*	2			1.75	.504	2.82	.063	2.34	.189	1.20	.835
	*Seq*	3					1.07	.892	0.59	.991	−0.55	.994
	*Seq*	4							−0.48	.997	−1.62	.590
	*Seq*	5									−1.14	.865
	*Sim*	1	1.48	.677	2.27	.215	2.25	.226	3.31	**.016**	3.62	**.006**
	*Sim*	2			0.79	.968	0.77	.973	1.82	.455	2.14	.275
	*Sim*	3					−0.03	1	1.03	.906	1.35	.756
	*Sim*	4							1.06	.897	1.38	.742
	*Sim*	5									0.32	1
Mummification rate	*Acol*	1	−0.53	.995	1.94	.386	−0.07	1	1.75	.503	3.49	**.009**
	*Acol*	2			2.44	.154	0.46	.997	2.25	.225	3.94	**.002**
	*Acol*	3					−2000	.348	−0.18	1	1.63	.580
	*Acol*	4							1.82	.461	3.55	**.008**
	*Acol*	5									1.82	.461
	*Baca*	1	−3.44	**.011**	−0.68	.984	−2.58	.112	−1.89	.418	−2.13	.281
	*Baca*	2			2.76	.074	0.85	.956	1.55	.633	1.30	.782
	*Baca*	3					−1.90	.407	−1.21	.833	−1.45	.696
	*Baca*	4							0.70	.982	0.45	.998
	*Baca*	5									−0.25	1
	*Lfab*	1	−2.49	.139	0.45	.998	−2.55	.121	−0.21	1	−0.69	.983
	*Lfab*	2			2.94	**.046**	−0.06	1	2.28	.212	1.80	.471
	*Lfab*	3					−3000	**.039**	−0.66	.986	−1.14	.863
	*Lfab*	4							2.34	.188	1.86	.432
	*Lfab*	5									−0.48	.997
	*Seq*	1	−8.09	**<.001**	−5.73	**<.001**	−2.90	.052	−5.13	**<.001**	−6.30	**<.001**
	*Seq*	2			2.52	.128	5.06	**<.001**	3.16	**.025**	1.42	.717
	*Seq*	3					2.69	.087	0.64	.988	−0.95	.932
	*Seq*	4							−2.09	.301	−3.43	**.011**
	*Seq*	5									−1.55	.634
	*Sim*	1	−1.05	.900	0.95	.933	−0.03	1	−0.70	.981	−1.20	.834
	*Sim*	2			2000	.352	1.02	.909	0.28	1	−0.22	1
	*Sim*	3					−0.97	.925	−1.59	.605	−2.09	.299
	*Sim*	4							−0.68	.984	−1.18	.846
	*Sim*	5									−0.48	.997
Aphid number	*Acol*	1	3.02	**.036**	1.49	.674	3.27	**.017**	3.98	**.002**	4.26	**.001**
	*Acol*	2			−1.53	.645	0.26	1	0.97	.927	1.24	.815
	*Acol*	3					1.79	.477	2.50	.133	2.77	.069
	*Acol*	4							0.71	.980	0.98	.922
	*Acol*	5									0.27	1
	*Baca*	1	4.38	**<.001**	0.76	.974	2.82	.060	1.74	.508	3.41	**.011**
	*Baca*	2			−3.62	**.006**	−1.55	.631	−2.64	.097	−0.97	.928
	*Baca*	3					2.06	.313	0.98	.923	2.65	.093
	*Baca*	4							−1.08	.887	0.59	.992
	*Baca*	5									1.67	.554
	*Lfab*	1	3.78	**.003**	0.35	.999	3.63	**.006**	0.18	1	3.24	**.019**
	*Lfab*	2			−3.43	**.011**	−0.15	1	−3.60	**.006**	−0.54	.994
	*Lfab*	3					3.28	**.017**	−0.17	1	2.89	.051
	*Lfab*	4							−3.45	**.010**	−0.39	.999
	*Lfab*	5									3.06	**.032**
	*NoWasp*	1	1.82	.458	0.20	1	2.05	.318	0.11	1	0.49	.996
	*NoWasp*	2			−1.63	.578	0.11	1	−1.71	.527	−1.36	.750
	*NoWasp*	3					1.86	.433	−0.08	1	0.29	1
	*NoWasp*	4							−1.94	.383	−1.57	.622
	*NoWasp*	5									0.37	.999
	*Seq*	1	6.35	**<.001**	6.10	**<.001**	3.90	**.002**	5.91	**<.001**	6.03	**<.001**
	*Seq*	2			−0.25	1	−2.45	.149	−0.44	.998	−0.32	1
	*Seq*	3					−2.20	.245	−0.19	1	−0.07	1
	*Seq*	4							2.01	.344	2.13	.279
	*Seq*	5									0.12	1
	*Sim*	1	2.63	.098	0.73	.977	2.54	.120	3.51	**.008**	4.47	**<.001**
	*Sim*	2			−1.90	.410	−0.09	1	0.88	.951	1.84	.446
	*Sim*	3					1.81	.464	2.77	.069	3.73	**.004**
	*Sim*	4							0.96	.928	1.92	.393
	*Sim*	5									0.96	.930
Plant size	*Acol*	1	−2.09	.301	0.53	.995	−0.49	.996	1.19	.842	0.25	1
	*Acol*	2			2.62	.101	1.60	.603	3.27	**.017**	2.33	.189
	*Acol*	3					−1.02	.909	0.65	.986	−0.29	1
	*Acol*	4							1.68	.549	0.74	.977
	*Acol*	5									−0.94	.935
	*Baca*	1	−3.23	**.019**	1.39	.732	1.64	.576	2.29	.206	0.61	.990
	*Baca*	2			4.62	**<.001**	4.87	**<.001**	5.52	**<.001**	3.85	**.003**
	*Baca*	3					0.25	1	0.90	.946	−0.78	.971
	*Baca*	4							0.65	.986	−1.02	.909
	*Baca*	5									−1.68	.549
	*Lfab*	1	−3.60	**.006**	2.37	.174	−0.78	.971	3.03	**.035**	0.53	.995
	*Lfab*	2			5.98	**<.001**	2.82	.061	6.63	**<.001**	4.13	**.001**
	*Lfab*	3					−3.15	**.025**	0.65	.986	−1.84	.443
	*Lfab*	4							3.81	**.003**	1.31	.779
	*Lfab*	5									−2.50	.133
	*NoWasp*	1	−2.20	.243	0.41	.998	−1.39	.732	1.56	.629	0.86	.955
	*NoWasp*	2			2.59	.108	0.90	.946	3.66	**.005**	3.01	**.036**
	*NoWasp*	3					−1.80	.469	1.15	.861	0.45	.998
	*NoWasp*	4							2.95	**.044**	2.25	.223
	*NoWasp*	5									−0.70	.982
	*Seq*	1	−3.93	**.002**	−2.74	.075	0.41	.998	0.86	.955	−0.33	.999
	*Seq*	2			1.19	.842	4.34	**<.001**	4.79	**<.001**	3.60	**.006**
	*Seq*	3					3.15	**.025**	3.60	**.006**	2.41	.160
	*Seq*	4							0.45	.998	−0.74	.977
	*Seq*	5									−1.19	.842
	*Sim*	1	−1.96	.369	1.76	.496	2.50	.133	1.72	.522	0.29	1
	*Sim*	2			3.72	**.004**	4.46	**<.001**	3.68	**.005**	2.25	.223
	*Sim*	3					0.74	.977	−0.04	1	−1.47	.682
	*Sim*	4							−0.78	.971	−2.21	.241
	*Sim*	5									−1.43	.707

Response	Contrast	*z* 1	*p* 1	*z* 2	*p* 2	*z* 3	*p* 3	*z* 4	*p* 4	*z* 5	*p* 5	*z* 6	*p* 6
*Contrasts between treatments for each generation*													
Mummy number	*Acol*–*Baca*	5.09	**<.001**	1.85	.351	0.85	.913	1.28	.707	−0.98	.864	−1.07	.819
	*Acol*–*Lfab*	0.77	.939	−1.62	.486	−2.41	.121	−2.25	.169	−4.67	**<.001**	−4.25	**<.001**
	*Acol*–*Seq*	5.33	**<.001**	−0.78	.935	−0.63	.970	1.35	.659	−1.36	.656	−3.54	**.005**
	*Acol*–*Sim*	1.37	.647	0.22	1	−0.52	.986	0.46	.991	−0.83	.920	−1.68	.453
	*Baca*–*Lfab*	−4.33	**<.001**	−3.47	**.007**	−3.26	**.013**	−3.53	**.005**	−3.69	**.003**	−3.18	**.016**
	*Baca*–*Seq*	0.24	.999	−2.63	.072	−1.48	.576	0.08	1	−0.38	.996	−2.47	.106
	*Baca*–*Sim*	−3.72	**.003**	−1.63	.480	−1.37	.649	−0.82	.924	0.15	1	−0.60	.975
	*Lfab*–*Seq*	4.56	**<.001**	0.84	.917	1.78	.392	3.60	**.004**	3.31	**.011**	0.71	.953
	*Lfab*–*Sim*	0.60	.974	1.84	.357	1.89	.328	2.71	.059	3.84	**.002**	2.58	.081
	*Seq*–*Sim*	−3.96	**.001**	1000	.856	0.11	1	−0.90	.897	0.52	.985	1.87	.341
Mummification rate	*Acol*–*Baca*	6.48	**<.001**	3.68	**.003**	3.61	**.004**	4.05	**.001**	2.70	.061	0.43	.993
	*Acol*–*Lfab*	2.59	.080	0.70	.956	0.97	.867	0.20	1	0.56	.981	−1.73	.419
	*Acol*–*Seq*	5.83	**<.001**	−1.61	.492	−1.59	.507	2.96	**.031**	−0.83	.921	−3.80	**.002**
	*Acol*–*Sim*	1.68	.451	1.18	.762	0.57	.980	1.72	.426	−0.75	.943	−2.88	**.038**
	*Baca*–*Lfab*	−3.89	**.002**	−2.98	**.029**	−2.80	**.047**	−3.86	**.002**	−2.27	.162	−2.50	.098
	*Baca*–*Seq*	−0.27	.999	−5.29	**<.001**	−5.52	**<.001**	−0.86	.909	−3.74	**.003**	−4.80	**<.001**
	*Baca*–*Sim*	−4.80	**<.001**	−2.50	.099	−3.23	**.014**	−2.33	.142	−3.49	**.006**	−3.75	**.003**
	*Lfab*–*Seq*	3.40	**.008**	−2.32	.148	−2.72	.058	2.77	.0500	−1.47	.584	−2.45	.111
	*Lfab*–*Sim*	−0.91	.893	0.48	.989	−0.43	.993	1.53	.548	−1.35	.660	−1.40	.631
	*Seq*–*Sim*	−4.25	**<.001**	2.80	**.047**	2.29	.157	−1.33	.671	0.03	1	0.99	.857
Aphid number	*Acol*–*Baca*	−2.27	.214	−1000	.916	−2.95	**.043**	−2.69	.084	−4.36	**<.001**	−3.06	**.032**
	*Acol*–*Lfab*	−2.42	.158	−1.70	.531	−3.47	**.009**	−2.09	.3000	−5.96	**<.001**	−3.36	**.013**
	*Acol*–*NoWasp*	−2.38	.171	−3.19	**.021**	−3.58	**.006**	−3.51	**.008**	−5.98	**<.001**	−5.89	**<.001**
	*Acol*–*Seq*	−2.62	.099	0.48	.997	1.68	.551	−2.04	.327	−0.83	.961	−0.97	.927
	*Acol*–*Sim*	−0.39	.999	−0.75	.975	−1.09	.883	−1.07	.891	−0.84	.960	−0.20	1
	*Baca*–*Lfab*	−0.15	1	−0.70	.982	−0.53	.995	0.60	.991	−1.60	.599	−0.31	1
	*Baca*–*NoWasp*	−0.11	1	−2.25	.224	−0.63	.988	−0.83	.962	−1.62	.585	−2.83	.059
	*Baca*–*Seq*	−0.35	.999	1.48	.675	4.62	**<.001**	0.65	.987	3.53	**.007**	2.09	.300
	*Baca*–*Sim*	1.88	.421	0.25	1	1.85	.436	1.62	.5900	3.52	**.008**	2.86	.055
	*Lfab*–*NoWasp*	0.04	1	−1.58	.611	−0.11	1	−1.43	.711	−0.02	1	−2.52	.125
	*Lfab*–*Seq*	−0.21	1	2.18	.252	5.15	**<.001**	0.05	1	5.13	**<.001**	2.39	.166
	*Lfab*–*Sim*	2.03	.333	0.95	.932	2.38	.171	1.01	.912	5.12	**<.001**	3.17	**.023**
	*NoWasp*–*Seq*	−0.24	1	3.65	**.005**	5.25	**<.001**	1.48	.6800	5.15	**<.001**	4.92	**<.001**
	*NoWasp*–*Sim*	1.99	.356	2.48	.136	2.49	.136	2.44	.1500	5.14	**<.001**	5.69	**<.001**
	*Seq*–*Sim*	2.23	.231	−1.23	.820	−2.77	.069	0.96	.928	−0.01	1	0.77	.972
Plant size	*Acol*–*Baca*	0.86	.956	−0.23	1	1.68	.550	2.89	.051	1.91	.400	1.21	.832
	*Acol*–*Lfab*	1.17	.850	−0.27	1	2.92	**.046**	0.90	.947	2.92	**.046**	1.44	.701
	*Acol*–*NoWasp*	1.95	.377	1.60	.598	1.83	.448	1.09	.884	2.30	.201	2.53	.121
	*Acol*–*Seq*	1.36	.748	−0.39	.999	−1.75	.498	2.22	.234	1.05	.899	0.82	.964
	*Acol*–*Sim*	−0.70	.981	−0.58	.992	0.47	.997	2.14	.271	−0.19	1	−0.66	.986
	*Baca*–*Lfab*	0.31	1	−0.04	1	1.25	.812	−1.99	.354	1.01	.913	0.23	1
	*Baca*–*NoWasp*	1.09	.884	1.82	.454	0.16	1	−1.79	.473	0.39	.999	1.33	.770
	*Baca*–*Seq*	0.51	.996	−0.16	1	−3.43	**.010**	−0.66	.986	−0.86	.956	−0.39	.999
	*Baca*–*Sim*	−1.56	.626	−0.35	.999	−1.21	.832	−0.74	.976	−2.11	.291	−1.87	.424
	*Lfab*–*NoWasp*	0.78	.971	1.86	.431	−1.09	.884	0.19	1	−0.62	.989	1.09	.884
	*Lfab*–*Seq*	0.19	1	−0.12	1	−4.68	**<.001**	1.33	.7700	−1.87	.424	−0.62	.989
	*Lfab*–*Sim*	−1.87	.424	−0.31	1	−2.46	.145	1.25	.812	−3.12	**.026**	−2.11	.291
	*NoWasp*–*Seq*	−0.58	.992	−1.97	.364	−3.59	**.006**	1.13	.868	−1.25	.812	−1.72	.524
	*NoWasp*–*Sim*	−2.65	.092	−2.15	.266	−1.36	.748	1.05	.899	−2.50	.133	−3.20	**.021**
	*Seq*–*Sim*	−2.07	.311	−0.19	1	2.22	.234	−0.08	1	−1.25	.812	−1.48	.677
